# Protective effect of *Irvingia gabonensis* stem bark extract on cadmium-induced nephrotoxicity in rats

**DOI:** 10.2478/intox-2014-0030

**Published:** 2015-03-04

**Authors:** Oluwafemi Adeleke Ojo, Basiru Olaitan Ajiboye, Babatunji Emmanuel Oyinloye, Adebola Busola Ojo, Olaide Ibiwumi Olarewaju

**Affiliations:** 1 Department of Chemical Sciences, Biochemistry Unit, Afe Babalola University Ado-Ekiti, Ekiti State, Nigeria; 2 Department of Biochemistry, Ekiti State University, Ado-Ekiti, Ekiti State, Nigeria

**Keywords:** cadmium, antioxidant, nephrotoxicity, *Irvingia gabonensis*, creatinine

## Abstract

Cadmium has been considered a risk factor for humans as it accumulates in body tissues, such as the liver, lungs, kidneys, bones, and reproductive organs. The aim of the present study was to evaluate the effect of Irvingia gabonensis (IG) against cadmium (Cd)-induced nephrotoxicity. The study was performed on twenty (20) male rats divided into four groups: control group, cadmium group (4 mg/kg/day, intraperitoneally), cadmium + extract (200 mg/kg body weight by oral gavage) and cadmium + extract (400 mg/kg body weight by oral gavage). Changes in the kidney biochemical markers, namely glutathione (GSH), superoxide dismutase (SOD), catalase (CAT), aminotransferase (ALT), aspartate aminotransferase (AST) activities and levels of malondialdehyde (MDA), urea, and creatinine were determined in serum. Histological examinations were monitored. Exposure to Cd lowered the activities of kidney antioxidants, while it increased LPO levels. Levels of all disrupted parameters were alleviated by co-administration of IG extract. The malondialdehyde concentration of the rats treated with 200 and 400 mg/kg body weight of the extract significantly decreased (*p<*0.05) compared with the untreated cadmium rats. Yet the creatinine concentration decreased significantly (*p<*0.05) when the cadmium animals treated with 200 and 400 mg/kg body weight of the extract were compared with the cadmium control. Furthermore, histological alterations in the kidney were observed in cadmium untreated rats and these were ameliorated in cadmium treated rats by co-administration of IG extract. IG showed apparent protective and curative effect on Cd-induced nephrotoxicity.

## Introduction

Cadmium (Cd) is a toxic heavy metal in the environment. It is a highly accumulative toxicant with very long biological half-life (Friberg *et al.*, 1986). Cd is not biodegradable and its levels in the environment are increasing due to industrial activities, thus human exposure to Cd is inevitable (Friberg *et al.*, 1986; Goering *et al.*, 1995). Acute Cd exposure produced toxicities to the lung, liver, testes, and brain, while chronic exposure to Cd often leads to renal dysfunction, anemia, osteoporosis, and bone fractures (Friberg *et al.*, 1986; Goering *et al.*, 1995; Klaassen *et al.*, 1999). Cd is a potent carcinogen in a number of tissues of rodents and is classified as a human carcinogen (Waalkes, 2003). Cd was reported to generate reactive oxygen species (ROS) causing oxidative damage in various tissues (Liu *et al.*, 2008).

Exposure to Cd via different routes was found to cause increased lipid peroxidation (LPO) in membranes of erythrocytes and tissues, such as kidney, liver, brain, and testes, with thiobarbituric acid reactive substances (TBARS) and hydroperoxides used as indicators of oxidative damage (Jahangir *et al.*, 2005; Eybl *et al.*, 2006; Swarup *et al.*, 2007). Intake of Cd results in utilization of glutathione (GSH) and protein binding sulfhydryl groups, and consequently enhances the levels of free radicals, such as hydrogen peroxide, hydroxide, and superoxide anions (Valko *et al.*, 2005). The liver and kidney are considered the organs most vulnerable to Cd toxicity (Abd-El-Baset & Abd El-reheem, 2008; Abd-El-Reheem & Zaahkcuk, 2007; Asagba *et al.*, 2004; Ognjanović *et al.*, 2010). Cd nephrotoxicity was reported to result from generating free radicals and thus inducing cell necrosis and apoptosis (Reyes *et al.*, 2002; El-Sharaky *et al.*, 2007).

Herbal and natural products represent some of the most common forms of complementary and alternative medicines (Graham *et al.*, 2005). Numerous studies have exhibited the antioxidant properties of several natural products against many toxic materials (Shati & Elsaid, 2009; Shati & Alamri, 2010; Shati *et al.*, 2011). *Irvingia gabonensis* Baill. ex Lanen. (Irvingiaceae) (IG) is a commercial and indigenous fruit tree of West and Central Africa and identified as the most important tree for domestication (Nangue *et al.*, 2011; Dienagha & Miebi, 2011). The plant occurs freely in many parts of Africa, is an extensively used tropical African tree, classified as a high priority species and a Non-Timber Forest Product. As there has been no scientific report on the protective or therapeutic effect of IG against Cd-induced toxicity in the kidney of intoxicated animals, this study aimed to evaluate the protective and therapeutic effects of the ethanolic extract of IG against nephrotoxicity induced by Cd in Wistar rats.

## Materials and methods

### Chemicals

Cadmium chloride was bought from a local chemist in Ibadan, Nigeria. Thiobarbituric acids (TBA) were purchased from Aldrich Chemical Co. (Milwaukee, WI, USA). Glutathione, hydrogen peroxide, 5, 5’-dithios-bis-2-nitrobenzoic acid (DNTB) and epinephrine were from Sigma Chemical Co., Saint Louis, MO USA. Trichloroacetic acid (TCA) and Thiobarbituric acid (TBA) were purchased from British Drug House (BDH) Chemical Ltd., Poole, UK. Other reagents were of analytical grade and the purest quality available.

### Collection and extraction of *Irvingia gabonensis* stem bark

The stem bark of *Irvingia gabonensis* was collected on 16^th^ January, 2014 in Ado-Ekiti (Ekiti State) and authenticated at the Department of Plant Science, Ekiti State University. The stem bark of *Irvingia gabonensis* was air-dried and crushed into fine powder. The powdered part extracted with ethanol using maceration and the extract was concentrated in vacuum at 40 °C with a rotary evaporator and water bath to dryness. The yield of the extraction was 5.01%.

### Preliminary phytochemical screening

The preliminary phytochemical screening was carried out with ethanolic extracts of *Irvingia gabonensis* stem bark for the detection of various phytochemicals. Tests for common phytochemicals were carried out by standard methods (Srinivasan *et al.*, 2007).

### Animals

Male Wistar rats (*Rattus norvegicus*) weighing between 80–120 g were bought from the animal house of the Department of Chemical Sciences, Biochemistry Unit, Afe Babalola University, Nigeria. The animals were kept in aired cages at room temperature (28–30 °C) and received normal laboratory chow (Ladokun Feeds, Ibadan, Nigeria) and water *ad libitum*.

### Ethical approval

Handling and treatments of rats were conform to the guidelines of the National Institute of Health (NIH publication 85-23, 1985) for laboratory animal care and use. The ethical committee of the Afe Babalola University approved this study. The use of all animals in this study followed the guidelines of the institutional Animal Ethical Committee given by the Committee for Control and Supervision of Experiments on Animals (CPCSEA).

### Induction of experimental animals

Cadmium was induced in groups II, III and IV. Briefly, Cadmium dissolved in distilled water was given by intravenous injection (through tail vein) at a dose of 4 mg/kg body weight.

### Study design

Twenty male rats were divided into four groups of five rats each. Group I – Control (distilled water); Group II–cadmium chloride (4 mg/kg b.w.); Group III – *Irvingia gabonensis* (200 mg/kg b.w.) (14 days) + cadmium chloride (4 mg/kg b.w.); Group IV – *Irvingia gabonensis* (400 mg/kg b.w.) (14 days) + cadmium chloride (4 mg/kg b.w.) according to the method of Ojo *et al.* ( 2014).

### Preparation of tissue homogenate

Kidney tissues were quickly removed, washed in ice-cold isotonic saline and blotted individually on ash-free filter paper. The tissues were then homogenized in 0.1 M 2-amino-2-(hydroxymethyl)-1,3-propanediol hydrochloride buffer, pH 7.4, using a Potter-Elvehjem homogenizer at 4 °C. The crude tissue homogenate was centrifuged at a speed of 9 000 rpm for 15 min in a cold centrifuge and the supernatant was kept at –20 °C for estimation of GSH, SOD and CAT activities.

### Preparation of serum

Blood collected from the heart of the animals into plain centrifuge tubes was allowed to stand for 1 h. Serum was prepared by centrifugation at 3 000 g for 15 min in a Beckman bench centrifuge. The clear supernatant was used for assessing the serum lipid profile and enzymes.

### Biochemical tests

Protein content of the samples was tested by the method of Lowry *et al.* (1951) using bovine serum albumin as standard. The alanine and aspartate aminotransferases (ALT and AST) were tested by the combined methods of Mohun and Cook, (1957) and Reitman and Frankel (1957). Lipid peroxidation level was tested by the reaction between 2-thiobarbituric acid (TBA) and malondialdehyde (MDA), a product of lipid peroxides as described by Buege and Aust, (1978). The tissue superoxide dismutase (SOD) was determined by the nitro blue tetrazolium (NBT) decrease method of McCord and Fridovich, (1969). Catalase (CAT) was assessed spectrophotometrically by measuring the rate of decomposition of hydrogen peroxide at 240 nm as described by Aebi (1974). Reduced glutathione level was determined by the method of Beutler *et al.* (1963). By this method a stable (yellow) color is developing when 5’,5’-dithiobis-(2-nitrobenzoic acid) (Ellman's reagent) is mixed to sulfhydryl compounds. The chromophoric product resulting from Ellman's reagent with reduced glutathione (2-nitro-5-thiobenzoic acid) holds a molar absorption at 412 nm, which is part of the reduced glutathione in the test sample. Glutathione peroxidase (GPx) was tested by the method of Rotruck *et al.* (1973). When this substance is mixed with reduced glutathione, its absorption shifts to a longer wavelength of 340 nm and increase at this wavelength provides a direct measurement of the enzymatic reaction.

### Determination of serum urea concentration

The concentration of serum urea was determined using the method of Tietz (1994) as outlined in Randox kits, UK.

### Determination of serum creatinine concentration

The concentration of serum creatinine was determined using the method of Tietz (1994) as outlined in Randox kits, UK.

### Histopathology of tissues

The kidney from control and experimental groups were fixed with 10% formalin, embedded in paraffin and cut into longitudinal sections of 5 µm thickness. The sections were stained with hematoxylin and eosin dye for histopathological observation.

### Statistical analysis

All the data are expressed in mean ± SEM. The significance of difference in means between control and treated animals was determined by one-way analysis of variance (ANOVA) followed by the Duncan multiple range test for analysis of biochemical data using SPSS (20.0). Values were considered statistically significant at *p<*0.05.

## Results

### Investigation of phytochemicals

The ethanolic extract was found to contain compounds known to have antioxidant activity, like tannins, phlobatannins, flavonoids, anthocyanin, cardiac glycosides and alkaloids (Table 1).

**Table 1:** Phytochemical screening of ethanolic extract of *Irvingia gabonensis* stem bark.

Phytochemical	Extract Content
Alkaloids	+++
Tannin	++
Phlobatannins	++
Saponin	+
Flavonoids	+++
Anthraquinones	++
Phenol	+++
Cardiac glycosides	++

+ = Trace amount present ++ = Moderate amount present, +++ = Noticeable amount present

### Effects of *Irvingia gabonensis* stem bark on body weight and relative weight of organs of cadmium-induced nephrotoxicity in rats

Table 2 shows significant increases (*p<*0.05) in the relative weight of the kidney of cadmium untreated rats when compared with the control, while treatment with *Irvingia gabonensis* stem bark (100 and 200 mg/kg) significantly decreased the relative weight of the kidney of cadmium-induced rats to values statistically comparable to the control (*p>*0.05). All these changes induced by cadmium intoxication restored significantly (*p<*0.05) to near normal levels on administration of *Irvingia gabonensis* stem bark.

**Table 2:** Changes in the body weight and relative weight of organs of Cadmium-induced nephrotoxicity in rats treated with ethanolic extract of Irvingia gabonensis.

Treatment	Body weight (g)		Weight of organs (g)	Relative weight of organs
	Initial	Final	Kidney	Kidney
Control	100.25±0.21	117.46±5.32	6.35±0.27	0.78±0.05
Cadmium untreated	112.08±1.12	128.10±4.96	5.24±0.60	1.35±0.08^*^
Cadmium + 200mg/kg	86.45±2.23	125.55±3.11	3.40±0.22	0.53±0.02M^**^
Cadmium + 400mg/kg	98.02±3.35	131.20±2.09	6.22±0.40	0.72±0.03^**^

Values are means ± S.D. of 5 animals per group, cadmium = at 4 mg/kg, cadmium Treated = *Irvingia gabonensis* at 200 mg/kg, cadmium treated = *Irvingia gabonensis* at 400 mg/kg

* significantly different from Control (*p<*0.05)

** significantly different from cadmium untreated (*p<*0.05).

### Effects of *Irvingia gabonensis* stem bark on antioxidant parameters and marker enzymes in cadmium-induced nephrotoxicity in rats

Administration of cadmium chloride significantly increased (*p<*0.05) serum and kidney lipid peroxidation (LPO) products measured as thiobarbituric acid reactive substances (Table 3). Treatment with *Irvingia gabonensis* extract completely ameliorated cadmium-chloride-induced increase in LPO. In cadmium-induced rats, the activities of kidney GSH, SOD and CAT as well as GPx decreased significantly relative to the control (Table 4). Excellent performance of the extract at 400 mg/kg reversed the adverse effect of cadmium chloride by normalizing this enzymic antioxidant. *Irvingia gabonensis* treatment to cadmium-treated groups caused a significant increase in GPx activities as well as a noticeable increase in GSH level. In cadmium-induced rats, serum ALT and AST were significantly increased (table 5) relative to the control. Treatment with *Irvingia gabonensis* resulted in significant protection of the kidney, as indicated by reductions in the elevated levels of ALT and AST. There was evidence of amelioration in the treated group.

**Table 3:** Changes in the levels of lipid peroxidation in cadmium-induced nephrotoxicity rats treated with ethanolic extract of *Irvingia gabonensis*.

Treatments	KIDNEY (µmol MDA/mg protein)	SERUM (µmol MDA/mg protein)
Control	6.05±0.02	6.82±0.08
Cadmium Untreated	7.89±0.05^*^	8.26±0.06^*^
Cadmium + 200 mg/kg	5.66±0.04^**^	5.83±0.76^**^
Cadmium + 400 mg/kg	5.98±0.02^**^	6.04±0.40^**^

Values are means ± S.E.M. of 5 animals per group, cadmium Treated = *Irvingia gabonensis* at 200 mg/kg, cadmium treated = *Irvingia gabonensis* at 400 mg/kg

* significantly different from control (*p<*0.05)

** significantly different from cadmium untreated (*p<*0.05).

**Table 4:** Changes in the levels of kidney antioxidant parameters in Cadmium-induced rats treated with ethanolic extract of *Irvingia gabonensis*.

Treatment	GSH	GPx	SOD	CAT
	(mg /g tissue)		(U/mg protein)	
Control	39.85±0.15	45.55±0.83	47.26±1.01	45.77±1.08
Cadmium untreated	20.22±0.41^*^	21.77±0.81^*^	29.42±0.78^*^	28.45±0.82^*^
Cadmium + 200 mg/kg	35.42±0.65^**^	38.20±0.65^**^	38.88±0.33^**^	38.29±0.61^**^
Cadmium + 400 mg/kg	37.05±0.37^**^	42.28±0.71^**^	43.21±1.20^**^	42.20±0.82^**^

Values are means ± S.E.M. of 5 animals per group, cadmium Treated = *Irvingia gabonensis* at 200 mg/kg, cadmium treated = *Irvingia gabonensis* at 400 mg/kg

* significantly different from control (*p<*0.05)

** significantly different from cadmium untreated (*p<*0.05).

**Table 5:** Changes in the activities of serum and kidney alanine and aspartate aminotransferases in Cadmium-induced nephrotoxicity rats treated with ethanolic extract of *Irvingia gabonensis*.

Treatments	KIDNEY (U/L)		SERUM (U/L)	
	AST	ALT	AST	ALT
Control	58.65±0.02	65.56±2.24	4.54±1.77	6.37±1.46
Cadmium untreated	22.42±2.38^*^	21.28±2.04^*^	8.39±0.56^*^	9.89±2.24^*^
Cadmium + 200 mg/kg	43.60±1.25^**^	44.20±1.44^**^	4.13±1.50^**^	5.78±1.38^**^
Cadmium + 400 mg/kg	50.02±0.45^**^	57.87±1.34^**^	4.01±1.42^**^	6.01±1.28^**^

Values are means ± S.E.M. of 5 animals per group, cadmium untreated group = at 4 mg/kg cadmium Treated = *Irvingia gabonensis* at 200 mg/kg, cadmium treated = *Irvingia gabonensis* at 400 mg/kg

* significantly different from control (*p<*0.05)

** significantly different from cadmium untreated (*p<*0.05).

### Effects of *Irvingia gabonensis* stem bark on serum protein, urea and creatinine in cadmium-induced nephrotoxicity in rats

There was a significant decrease in the levels of serum total protein in the Cd group when compared with the control group (Table 6). However, levels of this compound in serum were significantly increased in IG + Cd rats when compared with the Cd control group. Levels of urea and creatinine in serum of the Cd group were significantly increased when compared with the control group (Table 6). Levels of serum urea and creatinine were significantly decreased in the IG + Cd group compared with the Cd group. The ameliorative effect of IG treatment on the levels of serum urea and creatinine was more prominent.

**Table 6:** Levels of total protein, urea and creatinine in the serum of control and experimental groups of rats.

Treatments	PROTEIN (g/dl)	UREA (mg/dl)	CREATININE (mg/dl)
Control	7.8±0.49	0.89±0.01	0.84±0.01
Cadmium untreated	4.48±0.02^*^	1.33±0.78^*^	1.24±0.04^*^
Cadmium + 200 mg/kg	6.89±0.45^**^	0.62±0.65^**^	0.58±0.43^**^
Cadmium + 400 mg/kg	7.2±0.32^**^	0.76±0.34^**^	0.68±0.02^**^

Values are means ± S.E.M. of 5 animals per group, cadmium chloride untreated group = at 4 mg/kg cadmium Treated = *Irvingia gabonensis* at 200 mg/kg, cadmium treated = *Irvingia gabonensis* at 400 mg/kg

* significantly different from control (*p<*0.05)

** significantly different from cadmium untreated (*p<*0.05).

### Effects of *Irvingia gabonensis* stem bark on kidney histology

**Figure 1:**
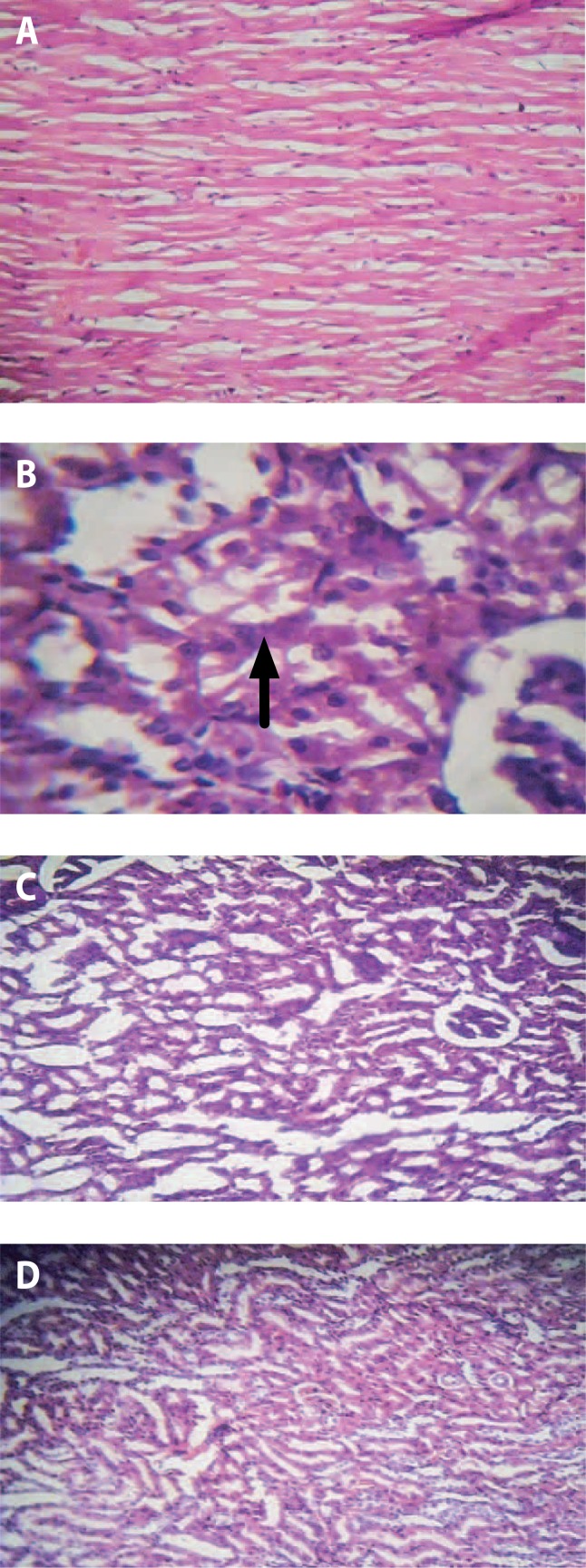
Changes in histology of kidney samples of cadmium-induced nephrotoxicity in rats treated with *Irvingia gabonensis* ethanolic stem bark extract. A: Control, B: Cadmium untreated, C: Cadmium + *I. gabonensis* (2 00mg/kg), D: Cadmium + *I. gabonensis* (400 mg/kg). Black arrow shows tubular degeneration, necrosis and severe renal cortical congestion.

Histology of the kidney slide of cadmium untreated rats showed tubular degeneration, necrosis and severe renal cortical congestion (Figure 1). Treatment with ethanolic extract of stem bark *Irvingia gabonensis* (200 and 400 mg/kg) confirmed the nephro-protective activity as a significant recovery of nephron damage and decreased necrosis was evident against cadmium-induced nephrotoxicity in the kidney of the rats, comparable to their control. The histological results further corroborated the biochemical findings suggesting the useful effects of *Irvingia gabonensis* stem bark in cadmium-induced toxicity in rats.

## Discussion

Cadmium is a well-known human carcinogen and a potent nephrotoxin. It is a potent inducer of oxidative stress and affects the cellular antioxidant defense potential bi-phasically by reserve and improvement of several antioxidant enzymatic and non-enzymatic molecules. The phytochemical study of *Irvingia gabonensis* stem bark extracts revealed the presence of polyphenol-rich compounds. Polyphenols have been suggested to decrease oxidative stress in humans. Flavonoids found in the extract may inhibit oxidative stress by scavenging free radicals, acting as reducing agents, hydrogen atom donating molecules or singlet oxygen quenchers, chelating metal ions and sparing other antioxidants (*e.g.* carotene, vitamin C and E) (Fuhrman and Aviram, 2001). The literature revealed that the carbonyl groups present in flavonoids and phenolic compounds were responsible for the antioxidant activity (Sajeesh *et al.*, 2011). This investigation found that *Irvingia gabonensis* contained pharmacologically active substance(s) such as alkaloids, glycosides, saponins, tannins, flavonoids and phenolic compounds, which are responsible for the antioxidant activity.

In the present study, the potent chelation therapy with *Irvingia gabonensis* stem bark examined against cadmium-induced nephrotoxicity in rats showed that the mean body weight of the cadmium-exposed group decreased with the increase in relative liver weight, which agrees with the findings of other authors (EI-demerdash *et al.*, 2009; Rahman *et al.*, 1998), suggesting that prolonged exposure to cadmium is accompanied by an increased risk of diabetes mellitus, which explains the weight loss in rats. Kaltreider *et al.* (2001) reported that exposure to low heavy metals was damaging to glucocorticoids. The glucocorticoid hormones play a role in glucose control as well as carbohydrate, lipid and protein metabolism. Glucocorticoid dysfunction is linked to weight gain or loss. In cadmium exposed rats treated with *Irvingia gabonensis*, the changed body weight and liver weight parameters recovered to near normal levels due to the antioxidant effects of *Irvingia gabonensis* stem bark.

Many studies have shown that cadmium induces oxidative damage by producing ROS (Liu *et al.*, 2008; Chen *et al.*, 2008) and decreasing biological activities of some antioxidant enzymes, such as SOD and CAT (Ikediobi *et al.*, 2004; Uchida *et al.*, 2004), which play an important role in the antioxidant profile and in scavenging free radicals. Cadmium has also been reported to cause damage to lipids and so to generate LPO (El-Sharaky *et al.*, 2007; Renugadevi & Prabu, 2010). This study also showed that exposure to cadmium led to an increase of LPO indicated by the elevation of TBARS levels, which was associated with a distinct decrease in the activity of the antioxidants SOD, CAT, and GSH in the kidney of the animals exposed to cadmium. SOD, CAT and GSH are essential parts of the cellular antioxidant defense system and they play an essential role in the protection against oxidative stress (Messaoudi *et al.*, 2009). It has been proposed that cadmium induces oxidative stress and LPO by depleting GSH or by inhibition of antioxidant enzymes (Messaoudi *et al.*, 2009). Moreover, cadmium was shown to exert a direct inhibitory effect on SOD and CAT activities via cadmium – enzyme interaction with a resultant perturbation of enzyme topography critical for catalytic activity (Messaoudi *et al.*, 2009; Casalino *et al.*, 2002). Our results showed that elevation of the administered cadmium resulted in a marked increase of nephric LPO. This was consistent with other reports on cadmium-induced findings in rats (Ognjanović *et al.*, 2010; Casalino *et al.*, 2002). The significant decrease in the activities of SOD and CAT in the kidneys of the cadmium groups compared to the control group may be attributed, in part, to an overwhelming oxidative modification of enzymatic proteins and biomembrane lipids by ROS, as evident by increased levels of LPO (Casalino *et al.*, 2002).

Moreover, the protective effects of this extract may be related to its ability to chelate or sequester cadmium via formation of cadmium-flavonoid complexes (el-Ashmawy *et al.*, 2005; Nedorostova *et al.*, 2009) as a result of many conditions including cell necrosis, improved or increased synthesis, and alterations in the permeability of the enclosing cell membrane (Gaskill *et al.*, 2005). Interestingly, these adverse effects were significantly attenuated by *Irvingia gabonensis* in the treated groups, indicating a prominent nephroprotective effect of *Irvingia gabonensis* against cadmium nephrotoxicity. The increased levels of serum AST and ALT in cadmium exposed rats indicate an increased permeability and damage and/or necrosis of the kidney. In our study, we found that the extract of *Irvingia gabonensis* at a dose of 400 mg/kg caused a significant decrease in the activities of serum AST, ALT, which further supports the beneficial effects of the extract of *Irvingia gabonensis* in cadmium-induced rats.

Serum total protein represents a complex mixture containing a number of components which differ in properties and function. Hypo-proteinemia, protein deficiency in plasma, may be partly due to dietary insufficiency with subsequent impairment of the protein synthetic machinery, or to excessive excretion (Chawla, 2003). In the present study, there was a significant decrease in serum total protein levels in the cadmium groups compared with the control group.

It has been postulated that increased levels of serum urea and creatinine are linked to kidney disease (Chawla, 2003). Urea is the main nitrogenous end product of protein catabolism. It represents 90% of the total urinary nitrogen excretion. In this study, the cadmium group showed a significant increase in serum urea and creatinine that might suggest the inability of the kidney to excrete these products, indicating an impairment of kidney function. These effects could be attributed to the changes in the threshold of tubular re-absorption, renal blood flow, and glomerular filtration rate (Bishop *et al.*, 2000). The damaging effect of cadmium on kidneys was described by some authors (Casalino *et al.*, 2002; Jurczuk *et al.*, 2004). Several studies showed increased urea concentrations in serum, indicating reduced glomerular filtration rate in cadmium-exposed rats (Satarug S, Moore, 2004; Noonan *et al.*, 2002). In addition, cadmium was also proposed to exert a direct toxic effect on the glomeruli, leading to decrease in urea and creatinine clearance (Noonan *et al.*, 2002). The results of the current study showed that the ethanol extract of *Irvingia gabonensis* had a protective effect against cadmium-induced kidney damage, as established by the significant decrease in urea and creatinine levels in the *Irvingia gabonensis* treated group compared with the cadmium group.

Histological examination revealed that cadmium intoxication caused abnormal ultra-structural changes in kidney tissue, including tubular degeneration, necrosis and severe renal cortical congestion. Regarding the histopathological observation, *Irvingia gabonensis* treatment of cadmium-induced nephrotoxicity in rats revealed that the observed pathological impairments caused by cadmium recovered significantly, indicating that *Irvinga gabonensis* is capable of preventing the nephron damage induced by cadmium. It is thus suggested that *Irvingia gabonensis* may inhibit Cd-induced kidney damage. However, further studies are necessary to find out the actual mechanism of action of phytochemicals and their doses in the presence of oxidative stress due to Cd intoxication.

## Conclusion

In conclusion, the ethanolic extracts of *Irvingia gabonensis* exhibited protective effects against Cd-induced nephrotoxicity. For populations exposed to Cd, the use of the ethanolic extract of *Irvingia gabonensis* could thus be recommended.
